# Syntheses and structures of *trans*-di­aqua­bis(propane-1,3-di­amine-κ^2^*N*,*N*′)nickel(II) salts of two isomeric naphthalene­disulfonates

**DOI:** 10.1107/S2056989026010236

**Published:** 2026-10-09

**Authors:** Philip J. Squattrito, Christopher G. Gianopoulos

**Affiliations:** a Department of Chemistry and Biochemistry Central Michigan University,Mount Pleasant Michigan 48859 USA; b Department of Chemistry and Biochemistry, University of Toledo, Toledo, Ohio 43606, USA; University of Buenos Aires, Argentina

**Keywords:** crystal structure, octa­hedral nickel complex, naphthalene­disulfonate, metal salt, hydrogen bonding

## Abstract

Aqueous reactions of nickel(II) nitrate, propane-1,3-di­amine, and either naphthalene-1,5-di­sulfonic acid or disodium naphthalene-2,6-di­sulfonate produced crystals of *trans*-di­aqua­bis­(propane-1,3-di­amine-κ^2^*N*,*N*′)nickel(II) naphthalene-1,5-di­sulfonate, (I), and *trans*-di­aqua­bis­(propane-1,3-di­amine-κ^2^*N*,*N*′)nickel(II) naphthalene-2,6-di­sulfonate, (II), both [Ni(C_3_H_10_N_2_)_2_(H_2_O)_2_](C_10_H_6_O_6_S_2_), in high yields. (I) crystallizes in the monoclinic space group *P*2_1_/*c* while (II) crystallizes in the triclinic space group *P*1. In both structures, the cation and anion rest on centers of inversion. The nickel cations display *trans* octa­hedral coordination of two water mol­ecules and two bidentate propane-1,3-di­amine mol­ecules with Ni—O/N bond distances in the ranges 2.0960 (9)–2.1205 (7) Å (I) and 2.1110 (7)–2.1181 (9) Å (II). The packing in both structures features alternating layers of complex cations and naphthalene­disulfonate anions held together by O—H⋯O and N—H⋯O hydrogen bonds between the water and amine groups of the ligands and the sulfonate groups of the anions. These are the first metal salts of naphthalene-1,5-di­sulfonate and naphthalene-2,6-di­sulfonate that contain propane-1,3-di­amine. The packing in (I) and (II) is compared to each other and to those found in other nickel salts of the anions.

## Abstract 2

Two new layered structures of *trans*-di­aquabis(propane-1,3-di­amine)­nickel(II) complexes with isomeric naphthalene­disulfonate counter-ions are presented.

## 1. Chemical context

Metal–organic framework (MOF) structures are of inter­est for a variety of potential applications including gas storage and drug delivery (Dey *et al.*, 2014 ▸). Construction of MOFs using sulfonate anions as linkers has been an active area of research for the past few decades (Shimizu *et al.*, 2009 ▸; Zhang & Fei, 2019 ▸). As part of our ongoing study of metal sulfonate structures (Doney *et al.*, 2025 ▸), we serendipitously prepared the new compounds (I) and (II) during attempts to prepare MOFs with both sulfonate and amine bridges. In the event, the structures are composed of discrete nickel complexes and naphthalene­disulfonate anions, and belong to a family of compounds first reported twenty-five years ago (Chen *et al.*, 2001 ▸).
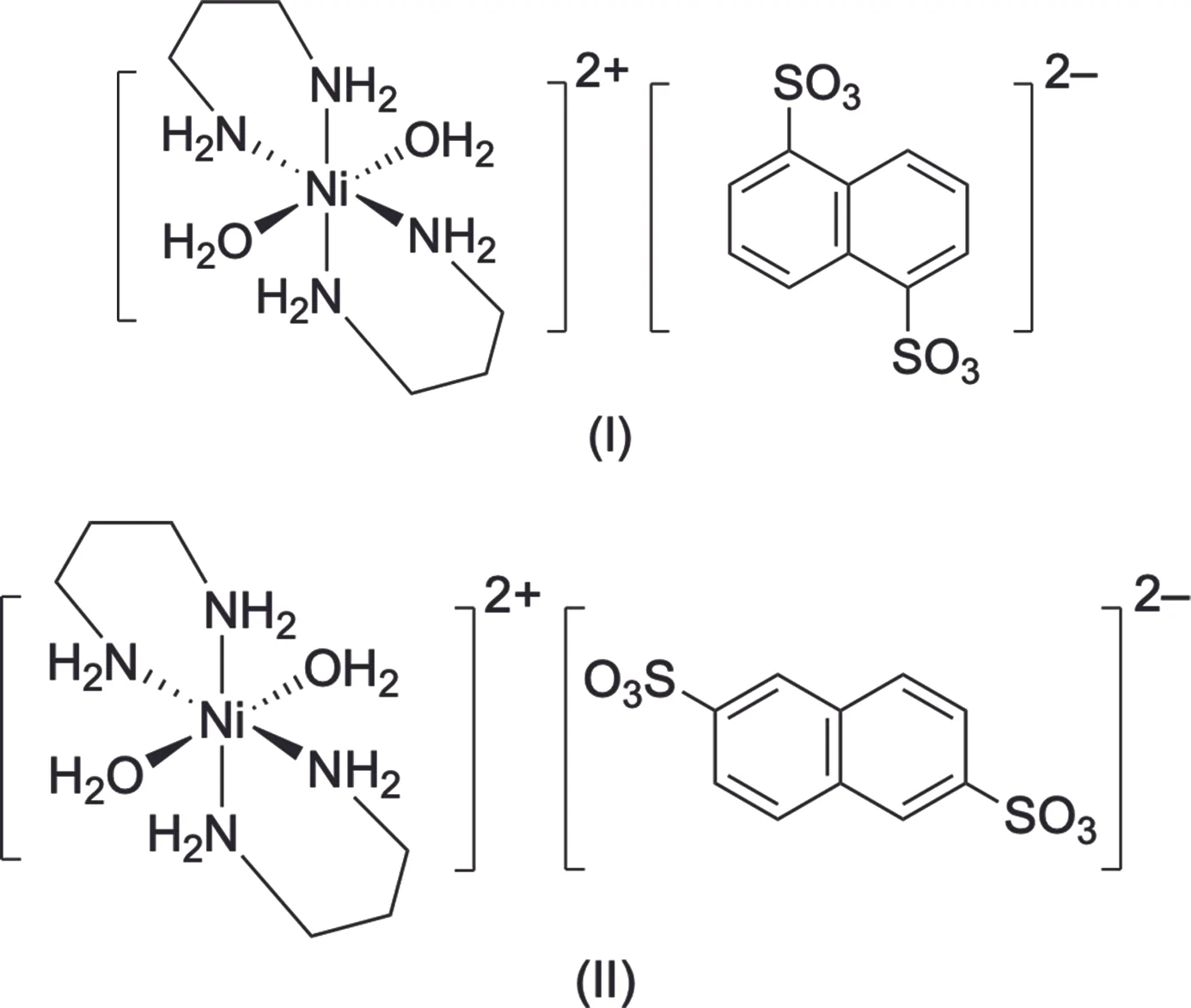


## 2. Structural commentary

**Figure 1:**
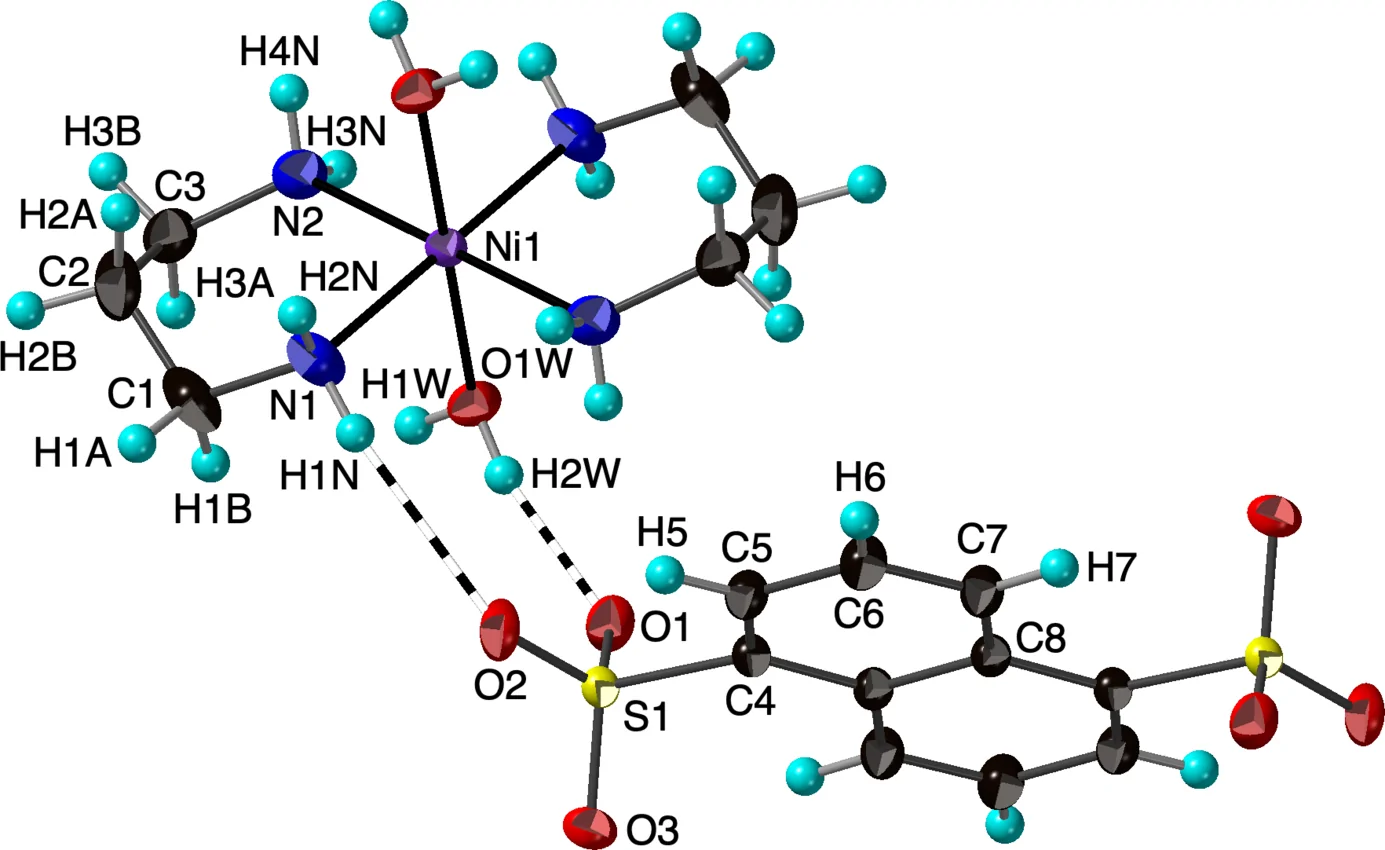
The mol­ecular structure of (I), showing the atom-numbering scheme. Displacement ellipsoids in this and subsequent figures are shown at the 70% probability level and hydrogen atoms are shown as small spheres of arbitrary radii. Hydrogen bonds are shown as striped cylinders. Symmetry-equivalent atoms are included to show the complete cation and anion.

The product of the reaction of nickel nitrate hexa­hydrate, propane-1,3-di­amine (DAP), and naphthalene-1,5-di­sulfonic acid is *trans*-[Ni(H_2_O)_2_(DAP)_2_](C_10_H_6_(SO_3_)_2_), (I). The compound crystallizes in the monoclinic space group *P*2_1_/*c* with the asymmetric unit consisting of half a [Ni(H_2_O)_2_(DAP)_2_]^2+^ cation and half a 1,5-C_10_H_6_(SO_3_)_2_^2–^ anion, both on centers of inversion. As a result of the symmetry, the nickel complex has a very regular *trans* octa­hedral coordination geometry (Fig. 1 ▸), with bond distances [Ni—O 2.1205 (7) Å, Ni—N 2.0961 (9), 2.1127 (9) Å] and angles [N—Ni—N 93.92 (4)^o^, N—Ni—O 91.08 (3), 88.67 (3)^o^] that are consistent with reported examples of this complex (Kim & Lee, 2002 ▸; Temel *et al.*, 2009 ▸; Rusanova, 2012 ▸). The naphthalene-1,5-di­sulfonate (1,5-nds) anion shows the pattern of slightly shorter (C4—C5, C6—C7 *ca*. 1.37 Å) and slightly longer (C5—C6, C7—C8, C8—C8^i^ [symmetry code: (i) −*x* + 2, −*y* + 1, −*z* + 1] *ca*. 1.40–1.42 Å) bond distances seen in [Ni(H_2_O)_6_](C_10_H_6_(SO_3_)_2_)] (Chen *et al.*, 2001 ▸). The sulfonate groups are positioned so that O2 nearly eclipses the ring [torsion angle O2—S1—C4—C5 = 3.66 (8)°. A similar eclipsed conformation is also seen in *trans*-di­aqua­bis­(ethylene­di­amine)nickel(II) naph­tha­lene-1,5-di­sul­fon­ate di­hy­drate (Chen *et al.*, 2001 ▸), perhaps indicating some resonance delocalization between the ring system and the sulfonate group. The cation and anion are linked by O—H⋯O and N—H⋯O hydrogen bonds between the water and amine groups and the sulfonate group (Table 1 ▸, Fig. 1 ▸).

**Figure 2:**
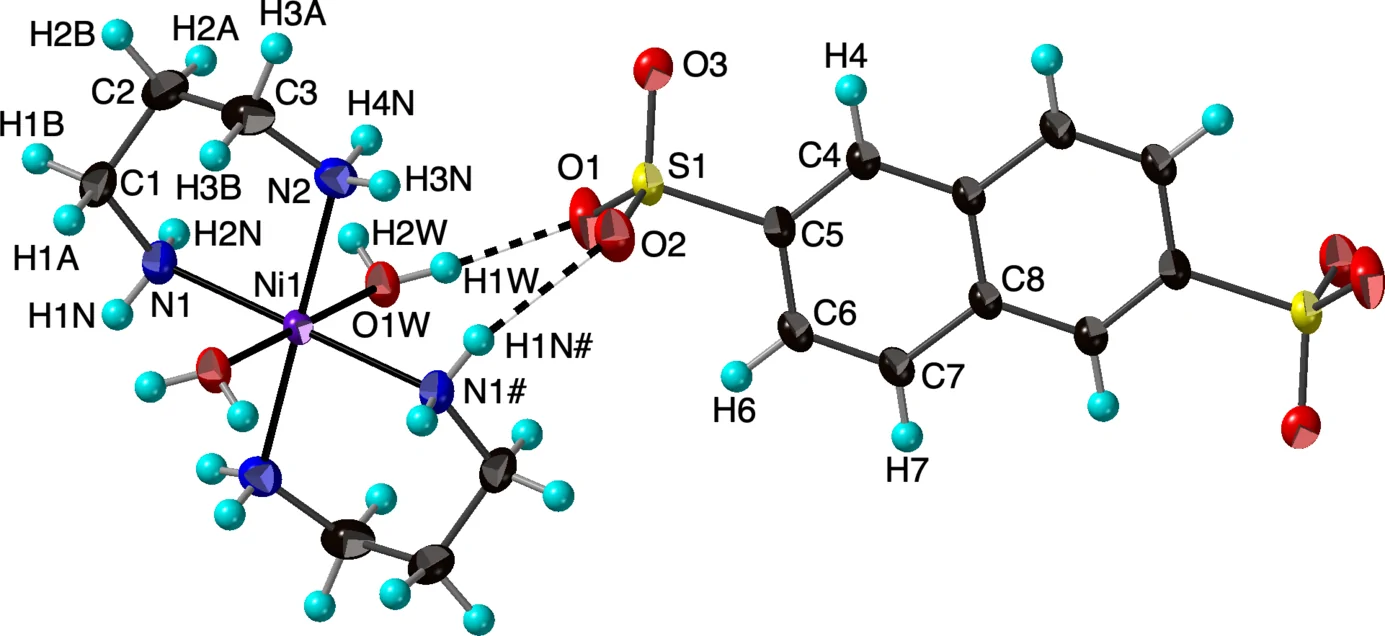
The mol­ecular structure of (II), showing the atom-numbering scheme. Hydrogen bonds are shown as striped cylinders. Symmetry-equivalent atoms are included to show the complete cation and anion. [Symmetry code: (#) 1 − *x*, 1 − *y*, 1 − *z*]

Compound (II), with the same chemical formula as (I), was the main product of the reaction of nickel nitrate hexa­hydrate, propane-1,3-di­amine (DAP), and the disodium salt of naphthalene-2,6-di­sulfonic acid. This compound crystallizes in the triclinic space group *P*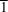 (the unit cell is coincidentally biclinic as the alpha angle is within two standard uncertainties of 90^o^) with half a [Ni(H_2_O)_2_(DAP)_2_]^2+^ cation and half a 2,6-C_10_H_6_(SO_3_)_2_^2–^ anion, both on centers of inversion, in the asymmetric unit. The *trans* octa­hedral coordination of the nickel ion [Ni—O 2.1110 (7) Å, Ni—N 2.1151 (8), 2.1181 (9) Å, N—Ni—N 90.30 (3)^o^, N—Ni—O 88.92 (3), 89.13 (3)^o^] is even more regular than in (I). The naphthalene-2,6-di­sulfonate (2,6-nds) anion shows the same pattern of C–C bond distances seen in (I). The sulfonate group is rotated a little over 10^o^ with respect to the ring relative to that in (I) [torsion angle O3—S1—C5—C4 = 15.34 (9)°]. Similar O—H⋯O water-sulfonate and N—H⋯O amine-sulfonate hydrogen bonds occur between the cation and anion (Table 2 ▸, Fig. 2 ▸).

Neither structure shows direct bonding between the Ni^2+^ cation and sulfonate O atoms. This is common for transition metal sulfonate salts, however, there are examples where 1,5-nds and 2,6-nds coordinate directly to Ni^2+^ as a terminal ligand (Gándara *et al.*, 2006 ▸) and 1,5-nds acts as a bridging ligand (Forbes & Sevov, 2009 ▸; Das *et al.*, 2015 ▸; Wei *et al.*, 2024 ▸), indicating its potential as a building block for MOF structures.

## 3. Supra­molecular features

**Figure 3:**
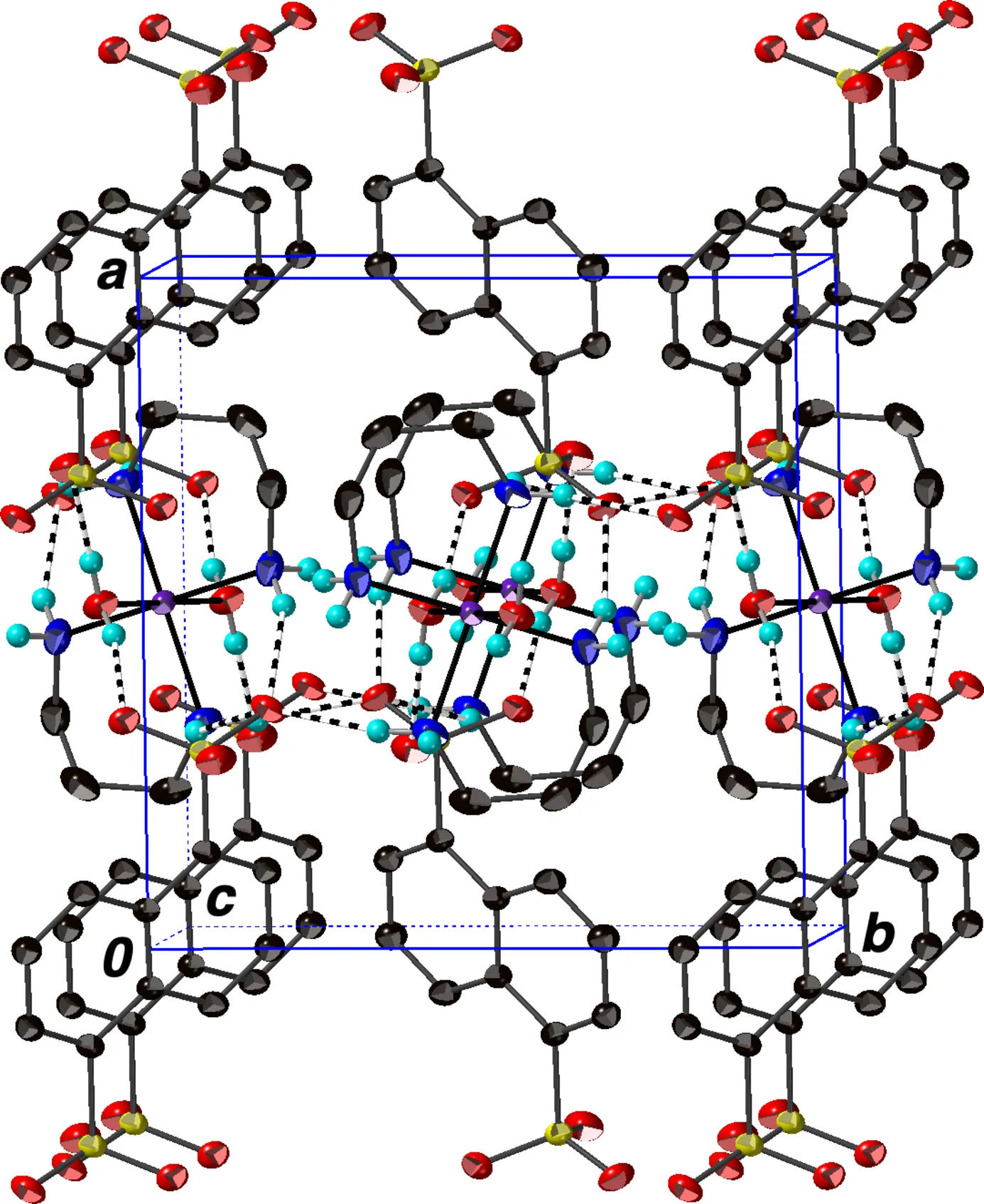
Packing diagram of (I) with the outline of the unit cell. The alternating layers of nickel complex cations and 1,5-nds anions are evident. O—H⋯O and N—H⋯O hydrogen bonds are shown as striped cylinders. H atoms bonded to C atoms have been omitted from all packing diagrams.

**Figure 4:**
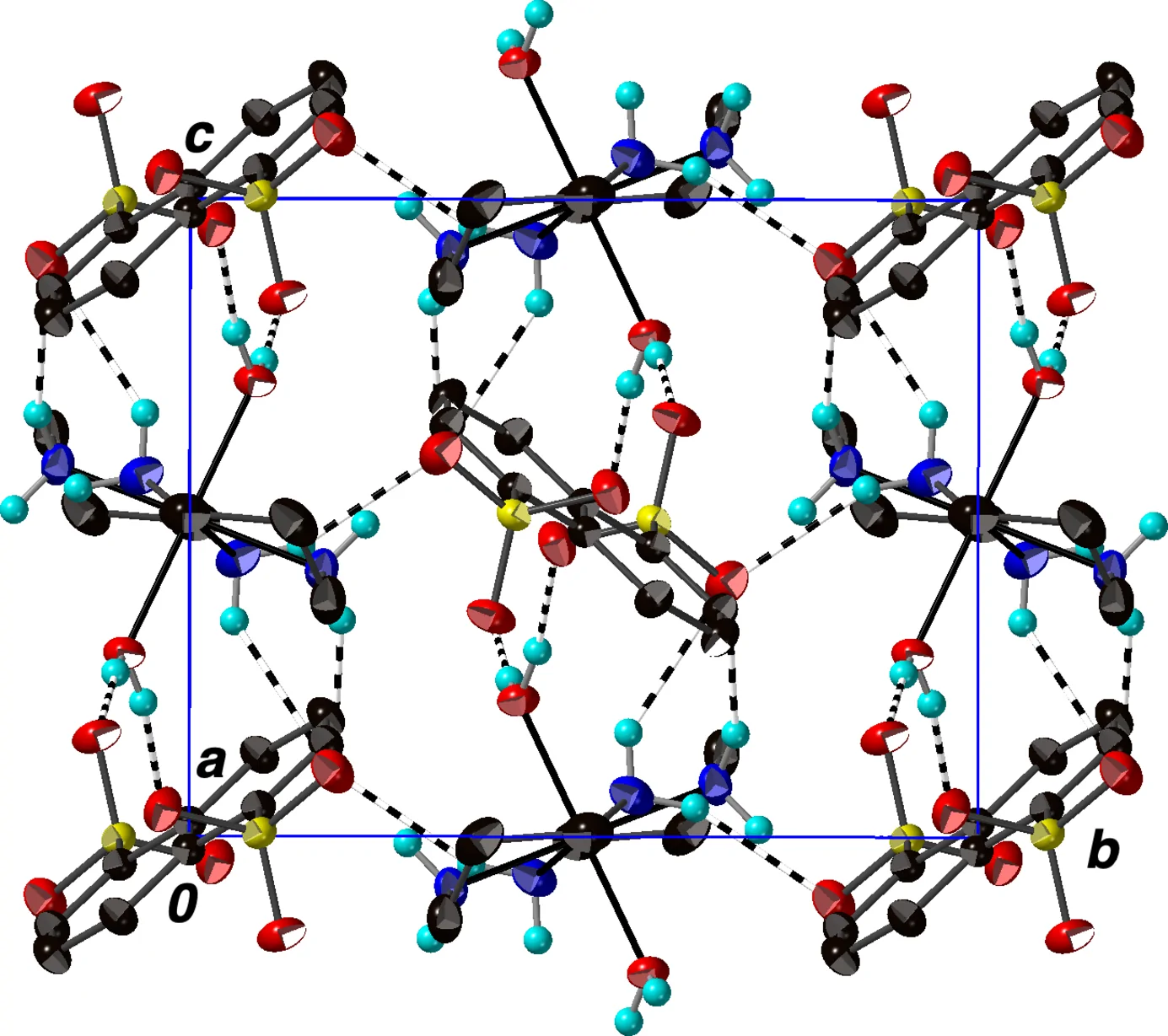
View of (I) perpendicular to the layers in the *bc* plane showing the alternating columns of cations and anions with opposite tilts of the nearest neighbors. The network of O—H⋯O and N—H⋯O hydrogen bonds (striped cylinders) is evident.

**Figure 5:**
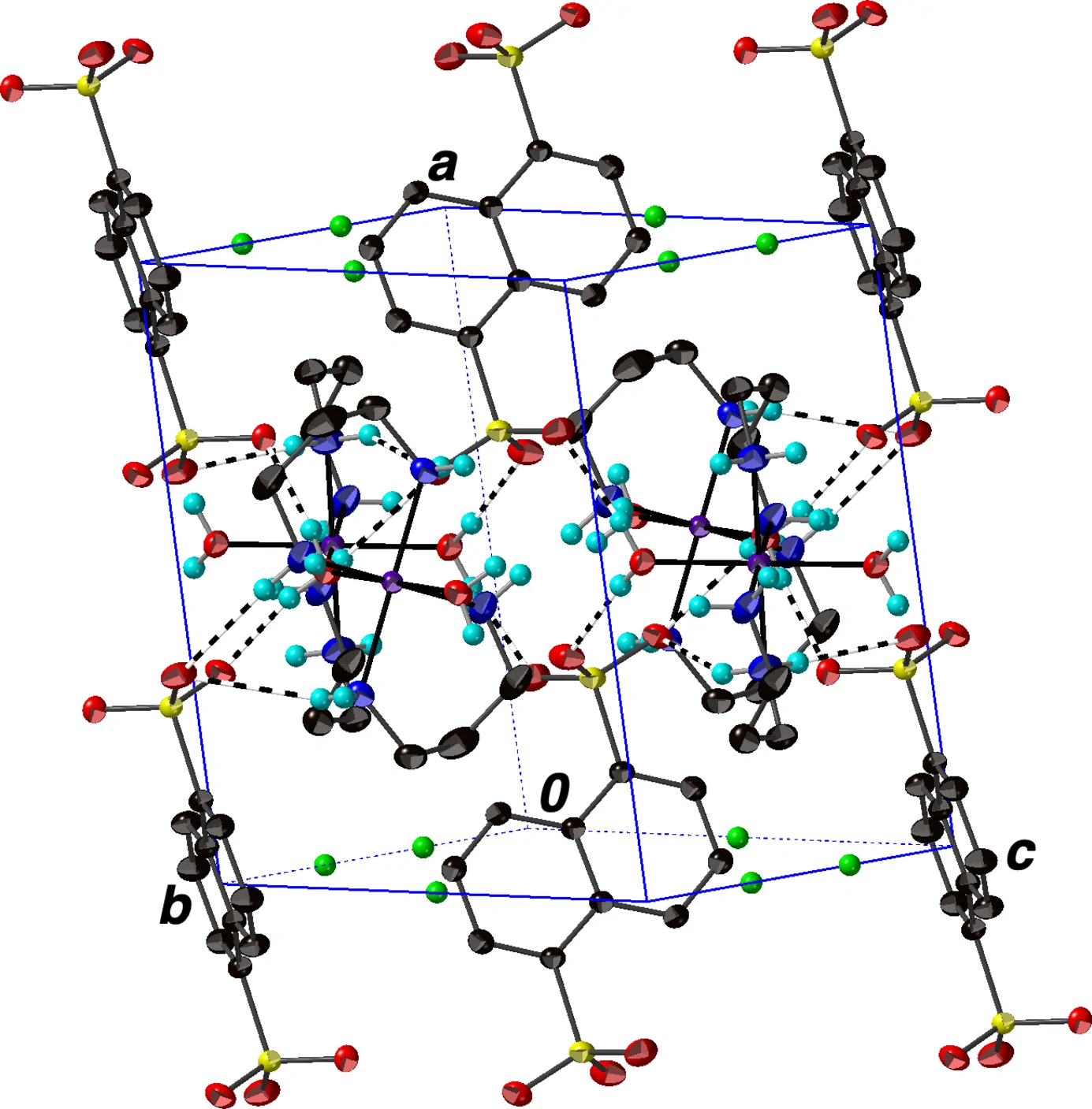
View of the packing in (I) showing the centroids (small green spheres) of cavities of radius *ca* 2.5 Å in the anion layer. Cavities in (I) and (II) were calculated using *CrystalMaker* (Palmer, 2014 ▸).

The nickel complex cations and 1,5-nds anions in (I) are segregated into layers parallel to the *bc* plane that stack in an alternating fashion along the *a*-axis direction (Fig. 3 ▸). Adjacent columns of cations and anions running along *b* and *c* are canted with respect to each other leading to a herringbone pattern in the *bc* plane (Fig. 4 ▸). The structure is anchored by a network of O—H⋯O and N—H⋯O hydrogen bonds between the coordinated water molecules and DAP ligands of the complexes and the sulfonate groups of the anions (Table 1 ▸). Unlike many structures containing aromatic ring systems in which parallel rings are packed *ca*. 3.4 Å apart to afford π stacking inter­actions, in (I) the 1,5-nds anions are more widely separated leading to cavities of *ca*. 2.5 Å radius between the 1,5-nds anions in the *bc* plane (Fig. 5 ▸) as calculated using *CrystalMaker* version 11.7.1 (Palmer, 2014 ▸).

The packing in (I) differs from that found in the closest related compound *trans*-di­aquabis(ethyl­enedi­amine)­nickel(II) 1,5-nds dihydrate (Chen *et al.*, 2001 ▸). In that compound, the naphthalene rings are all parallel to the *ac* plane, but the anions are not segregated from the cations in this or any of the planes corresponding to the faces of the unit cell. Rather, there are alternating layers of cations and anions parallel to (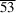 53 24) with the naphthalene rings slanted *ca*. 40^o^ relative to the stacking direction. Owing to this slant, the parallel ring systems are not face to face, and have ethyl­enedi­amine groups in between. The cavities seen in (I) are absent and the largest existing cavities are significantly smaller (radius *ca*. 1.8 Å). Evidently the hydrogen-bonding network including non-coordinated water mol­ecules is sufficiently different to drive the changes. Nevertheless, the density of (I) (1.596 Mg m^−3^) is actually about 2.2% larger than the ethyl­enedi­amine analog (1.560 Mg m^−3^), so the packing in (I) is not more open. For comparison, [Ni(H_2_O)_6_](1,5-nds), in which the parallel naphthalene rings are closer together and do not have groups from the ligands between them, has a calculated density of 1.795 Mg m^−3^ (Chen *et al.*, 2001 ▸). Another related di­aquabis(di­amine) complex salt, *trans*-di­aquabis(*o*-phenyl­enedi­amine)­nickel(II) 1,5-nds (Suyunov *et al.*, 2023 ▸) has alternating planes of cations and anions parallel to (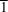
01) with the naphthalene rings only slanted about 12^o^ relative to the stacking direction. In this case, the phenyl groups on the ligands project into the naphthalene­disulfonate layers, so the segregation is not as great as in (I).

**Figure 6:**
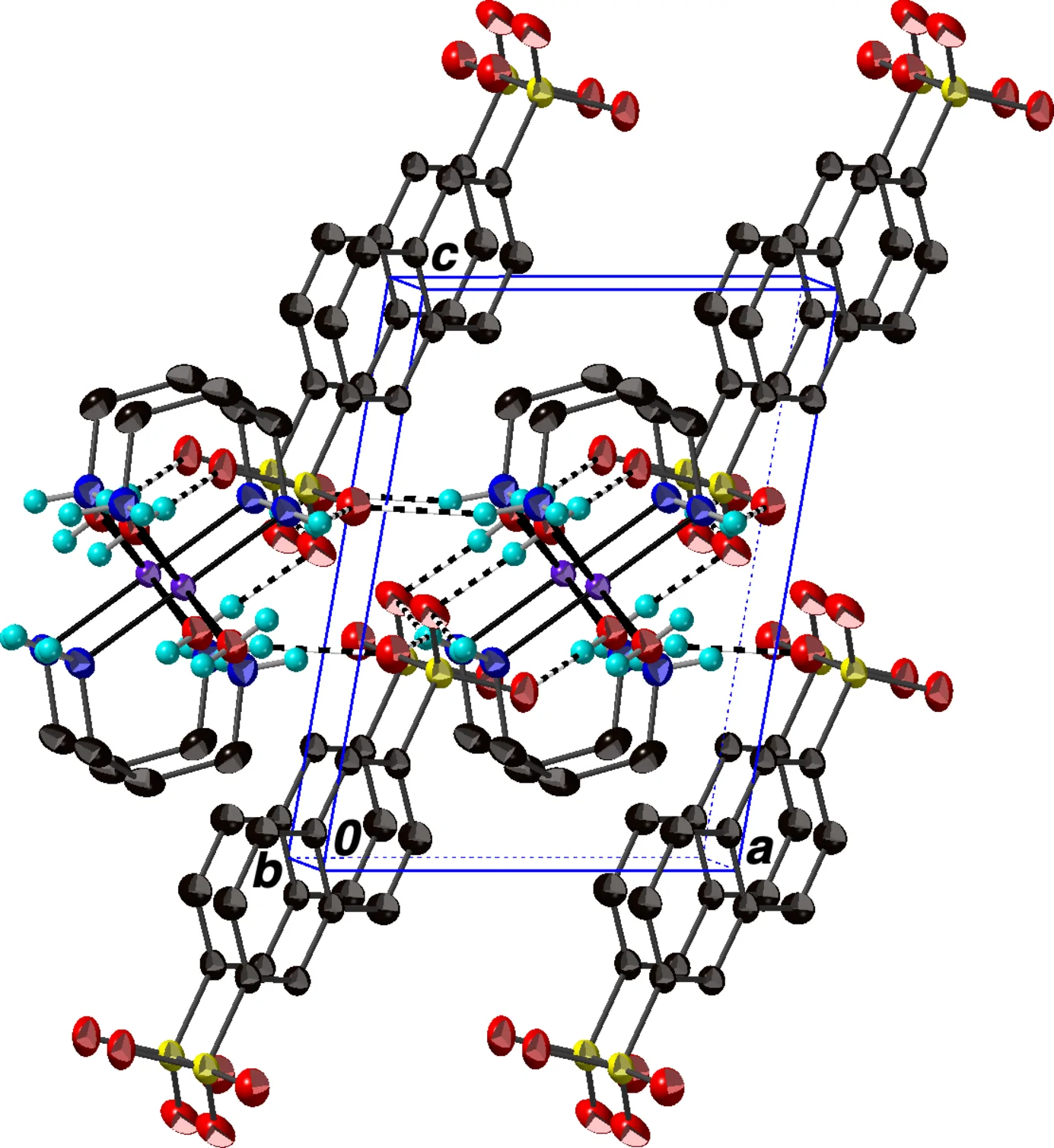
Packing diagram of (II) with the outline of the unit cell. The alternating layers of nickel complex cations and 2,6-nds anions are evident. O–H⋯O and N–H⋯O hydrogen bonds are shown as striped cylinders.

**Figure 7:**
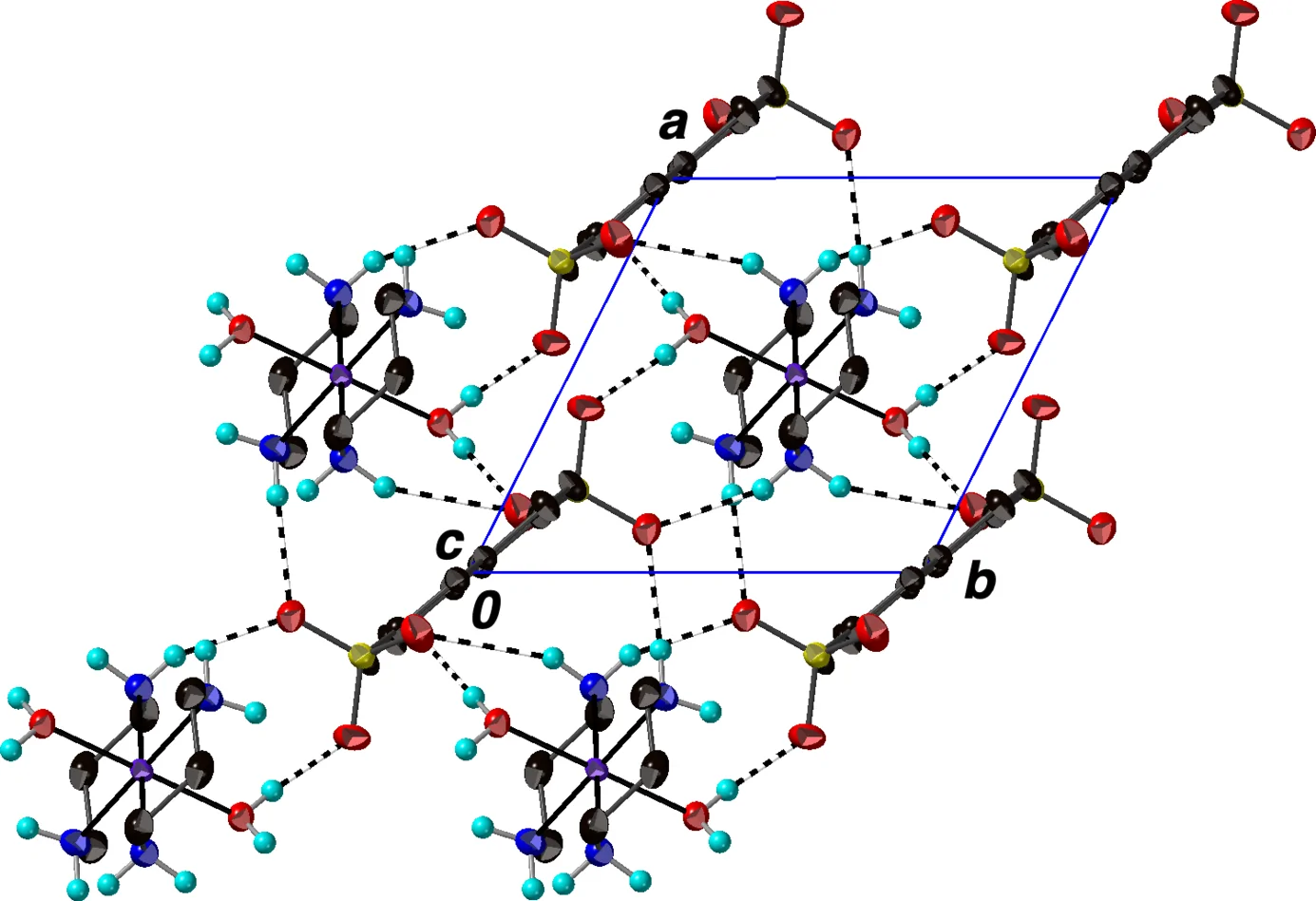
View of (II) perpendicular to the layers in the *ab* plane showing the alternating columns of cations and anions linked by the network of O—H⋯O and N—H⋯O hydrogen bonds (striped cylinders).

**Figure 8:**
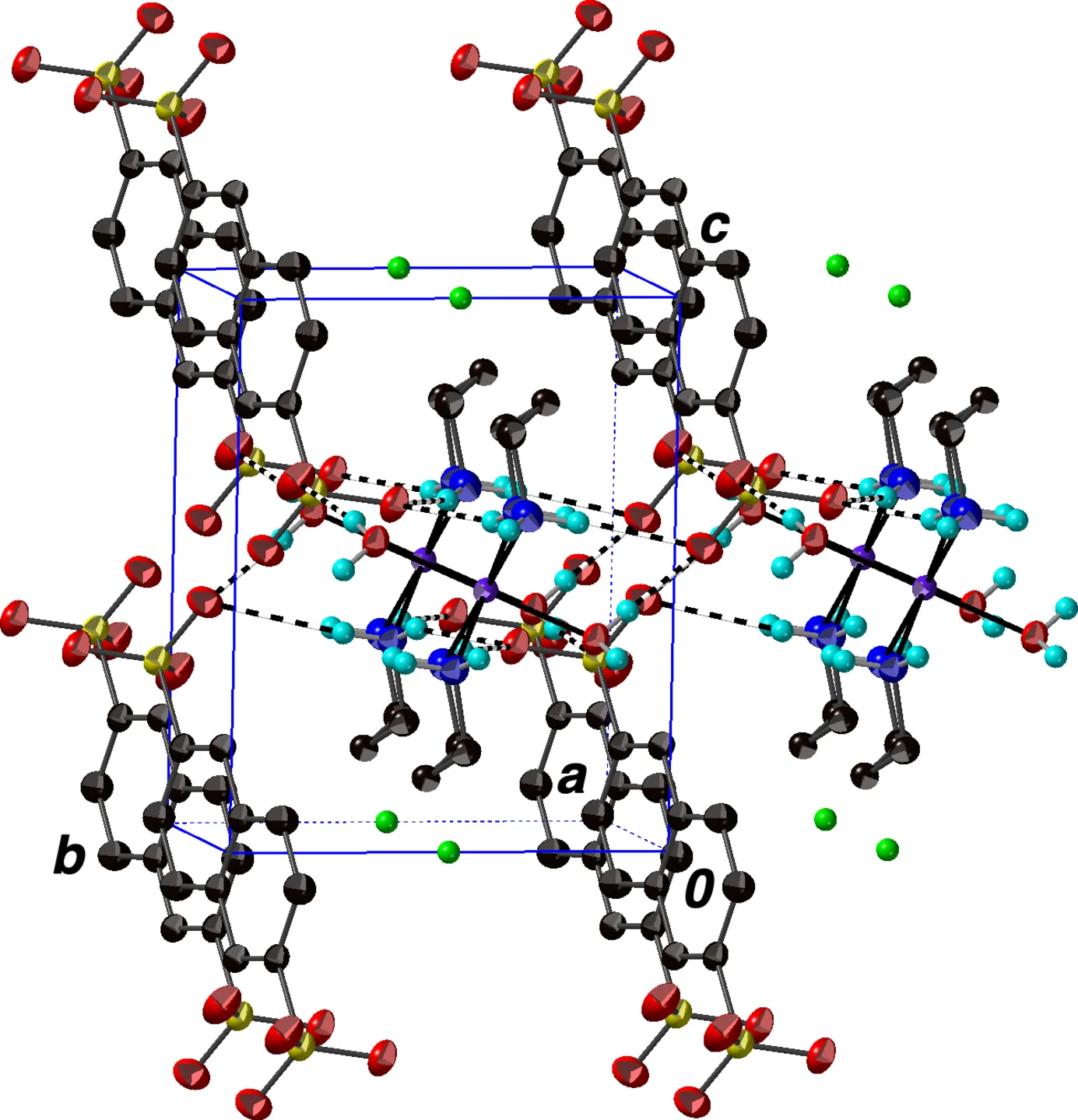
View of the packing in (II) showing the centroids (small green spheres) of cavities of radius *ca.* 2.5 Å in the anion layers.

Similar to (I), the cations and anions in (II) are segregated into layers parallel to one of the faces of the unit cell (*ab*) that stack in an alternating fashion in the direction of the remaining axis (*c*) (Fig. 6 ▸). All of the naphthalene rings are parallel to each other and there is an additional segregation of the cations and anions in the *bc* plane with alternating stacking along *a* (Fig. 7 ▸). Due to the dominant role of the sulfonate groups in the cation-anion inter­actions, the 2,6-nds anions are oriented with the long dimension of the ring system approximately along the stacking direction *c*, in contrast to the case in (I). A robust network of O—H⋯O and N—H⋯O hydrogen bonds link the cations and anions in the *ab* plane (Fig. 7 ▸, Table 2 ▸). As a consequence of the insertion of the DAP groups between them, the 2,6-nds anions are separated from each other sufficiently to result in cavities of radius *ca*. 2.5 Å centered at 0,1/2,0 between them (Fig. 8 ▸) (Palmer, 2014 ▸). The density of (II) (1.588 Mg m^−3^) is within 0.5% of the density of (I), indicating the structures are essentially equally open.

The most closely related compound to (II) with a reported structure is *trans*-di­aqua­bis­(*N*,*N′*-di­methyl­ethylenedi­amine)­nickel(II) 2,6-nds (Lian & Li, 2007 ▸). In this compound there is no clean segregation of the cations and anions with the long dimension of the naphthalene rings approximately along the stacking direction as is seen in (II) (Fig. 6 ▸). The only such segregation that occurs has the naphthalene rings approximately in the plane of the anion layer. Although there is a similar assortment of hydrogen bonds in all of these nickel(II) complex naphthalene­disulfonate structures, it appears that the details of the packing arrangement are highly sensitive to the size and shape of the amine ligand and the extent to which it protrudes into the anion layer.

## 4. Database survey

A search of the Cambridge Structural Database (CSD, Version 6.01, update of November 2025; Groom *et al.*, 2016 ▸) for naphthalene-1,5-di­sulfonate (1,5-nds) with any metal yielded 283 hits, of which 23 contain nickel. These comprise sixteen unique structures in which the 1,5-nds is a counter-ion only, one in which it is a terminal ligand to a single Ni^2+^ ion, and four in which it bridges two Ni^2+^ ions. Examples of the first category include hexa­aqua­nickel(II) 1,5-nds (refcode LUMVAQ; Chen *et al.*, 2001 ▸), *trans*-di­aqua­bis­(ethyl­enedi­amine)­nickel(II) 1,5-nds dihydrate (refcode LUMVEU; Chen *et al.*, 2001 ▸), bis­[bis­(2-amino­eth­yl)amine]­nickel(II) 1,5-nds dihydrate (refcode LUMVOE; Chen *et al.*, 2001 ▸), di­aqua­[tris­(2-amino­eth­yl)amine]­nickel(II) 1,5-nds monohydrate (refcode LUMVIY; Chen *et al.*, 2001 ▸), *trans*-tetra­aqua­bis­(imidazole)­nickel(II) 1,5-nds (refcode HIVDAS; Liu & Deng, 2008 ▸), tetra­aqua­(1,10-phenanthroline)nickel(II) 1,5-nds dihydrate (refcode KERBEP01; Gándara *et al.*, 2006 ▸), di­aqua­bis­(benzene-1,2-di­amine)­nickel(II) 1,5-nds (refcode LIWBEC; Suyunov *et al.*, 2023 ▸), and tetra­aqua­bis­(isonicotinamide)­nickel(II) 1,5-nds bis­(isonicotinamide) dihydrate (refcode TESDEC; Lian, 2012 ▸). The structure in which 1,5-nds bonds to a single Ni^2+^ ion through one of the sulfonate groups is aqua­(naphthalene-1,5-di­sulfonato-*O*)bis­(1,10-phenanthroline) nickel(II) (refcode GESVEG; Gándara *et al.*, 2006 ▸). The four structures in which a 1,5-nds anion bridges two Ni^2+^ ions through separate sulfonate groups are *catena*-[*cis*(μ_2_-naphthalene-1,5-di­sulfonato-*O*,*O′*)*tetra­kis*(imidazole)­nickel(II)] (refcode BUCTID; Forbes & Sevov, 2009 ▸), (μ-naphthalene-1,5-di­sulfonato)­hexa­aqua­bis­(4,4′-dimethyl-2,2′-bi­pyridine)­di­nickel(II) naphthalene-1,5-di­sulfonate dihydrate (refcode KONFOM; Wei *et al.*, 2024 ▸), *catena*-(tetra­kis­{μ-[3,5-bis­(2-amino­eth­yl)-1,3,5-triazinan-1-yl]methano­lato}tetra­kis­(μ-hy­droxo)bis­(μ-naphthalene-1,5-di­sulfonato)­tetra­aqua­octo­nickel(II) bis­(naphthalene-1,5-di­sulfonate) docosa­hydrate (refcode KUTSUP; Das *et al.*, 2015 ▸), and *catena*-[(μ_2_-naphthalene-1,5-di­sulfonato-*O*,*O′*)(1,4,8,11-tetra­aza­cyclo­tetra­dec­ane-*N*,*N′*,*N"*,*N′′′*)nickel(II) hydrate] (refcode UKESUY; Cai *et al.*, 2003 ▸). None of the 283 hits also contain propane-1,3-di­amine.

A search of the Cambridge Structural Database (CSD, Version 6.01, update of November 2025; Groom *et al.*, 2016 ▸) for naphthalene-2,6-di­sulfonate (2,6-nds) with any metal yielded 109 hits, of which ten contain nickel. These comprise nine structures in which the 2,6-nds is a counter-ion only and one in which it is a terminal ligand to a single Ni^2+^ ion. The first category includes tri­aqua­tris­(1*H*-imidazole)­nickel(II) 2,6-nds monohydrate (refcode RAHWEE; Zhang *et al.*, 2011 ▸), di­aqua­bis­(ethyl­enedi­amine)­copper(II) bis­[tris­(ethyl­enedi­amine)­nickel(II)] tris­(2,6-nds) tetra­hydrate (refcode TIJGAU; Cai *et al.*, 2001 ▸), *catena*-[(μ_2_-4,4′-bi­pyridine)­tetra­aqua­nickel(II) 2,6-nds tetra­hydrate] (refcode TUCLOT; Kanoo *et al.*, 2009 ▸), *trans*-di­aqua­bis­(*N*,*N′*-di­methyl­enedi­amine)­nickel(II) 2,6-nds (refcode XEYZEH; Lian & Li, 2007 ▸), and tris­(ethyl­enedi­amine)­nickel(II) 2,6-nds monohydrate (refcode YAFSOO; Huo *et al.*, 2004 ▸). The one structure in which 2,6-nds bonds to a single Ni^2+^ ion through one of the sulfonate groups is aqua­(naphthalene-2,6-di­sulfonato-*O*)bis­(1,10-phenanthroline) nickel(II) dihydrate (refcode GESVOQ; Gandara *et al.*, 2006 ▸). None of the 109 hits also contains propane-1,3-di­amine.

## 5. Synthesis and crystallization

For (I), 0.893 g (3.07 mmol) Ni(NO_3_)_2_^.^6H_2_O (Aldrich) was dissolved in 10 ml of water to give a clear green solution. A 1.126 g (3.12 mmol) sample of naphthalene-1,5-di­sulfonic acid tetra­hydrate (Aldrich, 97%) was dissolved in 50 ml of water to give a clear colorless solution. These were combined with stirring and the resulting solution was clear pale green. Approximately 0.75 ml (*ca.* 9 mmol) of propane-1,3-di­amine (Aldrich, 99%) was added dropwise over several minutes. The color changed gradually to blue and the solution remained clear. It was set out in a porcelain evaporating dish. Many purple crystals started forming after about one h. The remaining solution was pale blue. A majority of the solvent was allowed to evaporate over a few days and the crystals were collected by suction filtration (1.5 g, 92% yield based on Ni).

For (II), 0.904 g (3.11 mmol) Ni(NO_3_)_2_^.^6H_2_O (Aldrich) was dissolved in 10 ml of water and 1.032 g (3.11 mmol) of naphthalene-2,6-di­sulfonic acid disodium salt (Aldrich, 97%) was dissolved in 50 ml of water to give clear green and pale yellow solutions, respectively. After combining with stirring, 0.75 ml (*ca*. 9 mmol) of propane-1,3-di­amine (Aldrich 99%) was added dropwise over several minutes. The solution’s color changed to blue and then bluish purple, but it remained clear. The solution was set out in a porcelain evaporating dish. Crystals started to grow in the bottom of the dish within a few hours. Following evaporation of the solvent, large purple crystals were collected by hand (1.5 g, 91% yield based on Ni).

## 6. Refinement

Crystal data, data collection and structure refinement details are summarized in Table 3 ▸. For both structures, hydrogen atoms bonded to carbon atoms were located in difference-electron density maps, constrained on idealized positions, and included in the refinement as riding atoms with C—H = 0.95 Å (aromatic) or 0.99 Å (aliphatic) and their *U*_iso_ constrained to be 1.2 times the *U*_eq_ of the bonding atom. Oxygen- and nitro­gen-bound hydrogen atoms were located in difference electron-density maps and refined with isotropic displacement parameters while the O—H and N—H distances were restrained to 0.84 (1) Å and 0.91 (1) Å, respectively.

## Supplementary Material

Crystal structure: contains datablock(s) I, II. DOI: 10.1107/S2056989026010236/vu2023sup1.cif

Structure factors: contains datablock(s) I. DOI: 10.1107/S2056989026010236/vu2023Isup2.hkl

Structure factors: contains datablock(s) II. DOI: 10.1107/S2056989026010236/vu2023IIsup3.hkl

CCDC references: 2592371, 2592370

Additional supporting information:  crystallographic information; 3D view; checkCIF report

## Figures and Tables

**Table 1:** Hydrogen-bond geometry (Å, °) for (I)

*D*—H⋯*A*	*D*—H	H⋯*A*	*D*⋯*A*	*D*—H⋯*A*
O1*W*—H2*W*⋯O3^i^	0.83 (1)	1.99 (1)	2.8095 (10)	173 (2)
O1*W*—H1*W*⋯O1	0.83 (1)	1.92 (1)	2.7324 (10)	167 (2)
N1—H1*N*⋯O2	0.91 (1)	2.51 (1)	3.3726 (13)	159 (1)
N2—H3*N*⋯O2^ii^	0.90 (1)	2.34 (1)	3.1817 (13)	157 (1)
N2—H4*N*⋯O2^iii^	0.88 (1)	2.60 (2)	3.3207 (12)	140 (1)

Symmetry codes: (i) 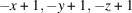; (ii) 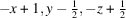; (iii) 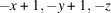.

**Table 2:** Hydrogen-bond geometry (Å, °) for (II)

*D*—H⋯*A*	*D*—H	H⋯*A*	*D*⋯*A*	*D*—H⋯*A*
O1*W*—H1*W*⋯O1	0.83 (1)	1.88 (1)	2.7065 (10)	174 (2)
O1*W*—H2*W*⋯O3^i^	0.82 (1)	1.92 (1)	2.7437 (11)	177 (2)
N1—H1*N*⋯O2^ii^	0.87 (1)	2.22 (1)	3.0039 (11)	150 (1)
N1—H2*N*⋯O3^i^	0.90 (1)	2.51 (1)	3.3444 (12)	155 (1)
N2—H3*N*⋯O2^iii^	0.90 (1)	2.18 (1)	3.0533 (12)	166 (1)

Symmetry codes: (i) 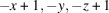; (ii) 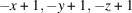; (iii) 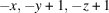.

**Table 3:** Experimental details

	(I)	(II)
Crystal data		
Chemical formula	[Ni(C_3_H_10_N_2_)_2_(H_2_O)_2_](C_10_H_6_O_6_S_2_)	[Ni(C_3_H_10_N_2_)_2_(H_2_O)_2_](C_10_H_6_O_6_S_2_)
*M*_r_	529.27	529.27
Crystal system, space group	Monoclinic, *P*2_1_/*c*	Triclinic, *P* 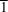
Temperature (K)	150	150
*a*, *b*, *c* (Å)	11.3312 (15), 10.9678 (15), 8.9430 (12)	7.9530 (3), 8.1103 (3), 9.7183 (3)
α, β, γ (°)	90, 97.745 (3), 90	89.9982 (11), 81.8907 (11), 63.368 (1)
*V* (Å^3^)	1101.3 (3)	553.32 (3)
*Z*	2	1
Radiation type	Mo *K*α	Mo *K*α
μ (mm^−1^)	1.12	1.12
Crystal size (mm)	0.62 × 0.34 × 0.22	0.44 × 0.20 × 0.12

Data collection		
Diffractometer	*APEX* duo with Photon II area detector	*APEX* duo with Photon II area detector
Absorption correction	Multi-scan (*SADABS*; Krause et al., 2015 ▸)	Multi-scan (*SADABS*; Krause *et al.*, 2015 ▸)
*T*_min_, *T*_max_	0.660, 0.747	0.660, 0.747
No. of measured, independent and observed [*I* > 2σ(*I*)] reflections	51023, 4915, 4314	29051, 4211, 3850
*R*_int_	0.033	0.028
(sin θ/λ)_max_ (Å^−1^)	0.822	0.769

Refinement		
*R*[*F*^2^ > 2σ(*F*^2^)], *wR*(*F*^2^), *S*	0.024, 0.063, 1.05	0.022, 0.058, 1.06
No. of reflections	4915	4211
No. of parameters	160	160
No. of restraints	6	6
H-atom treatment	H atoms treated by a mixture of independent and constrained refinement	H atoms treated by a mixture of independent and constrained refinement
Δρ_max_, Δρ_min_ (e Å^−3^)	0.49, −0.45	0.46, −0.40

Computer programs: *APEX3* and *SAINT* (Bruker, 2015 ▸), *SHELXT2018/2* (Sheldrick, 2015*a* ▸), *SHELXL2025/1* (Sheldrick, 2015*b* ▸), and *CrystalMaker* (Palmer, 2014 ▸).

## supplementary crystallographic information

### trans-Diaquabis(propane-1,3-diamine-κ2N,N')nickel(II) naphthalene-1,5-disulfonate (I) Crystal data

**Table:**

[Ni(C_3_H_10_N_2_)_2_(H_2_O)_2_](C_10_H_6_O_6_S_2_)	*F*(000) = 556
*M**_r_* = 529.27	*D*_x_ = 1.596 Mg m^−^^3^
Monoclinic, *P*2_1_/*c*	Mo *K*α radiation, λ = 0.71073 Å
*a* = 11.3312 (15) Å	Cell parameters from 9842 reflections
*b* = 10.9678 (15) Å	θ = 2.6–35.0°
*c* = 8.9430 (12) Å	µ = 1.12 mm^−^^1^
β = 97.745 (3)°	*T* = 150 K
*V* = 1101.3 (3) Å^3^	Parallelepiped, violet
*Z* = 2	0.62 × 0.34 × 0.22 mm

### trans-Diaquabis(propane-1,3-diamine-κ2N,N')nickel(II) naphthalene-1,5-disulfonate (I) Data collection

**Table:**

APEX duo with Photon II area detector diffractometer	4314 reflections with *I* > 2σ(*I*)
Radiation source: fine-focus sealed tube	*R*_int_ = 0.033
φ and ω scans	θ_max_ = 35.7°, θ_min_ = 2.6°
Absorption correction: multi-scan (SADABS; Krause et al., 2015)	*h* = −18→18
*T*_min_ = 0.660, *T*_max_ = 0.747	*k* = −17→17
51023 measured reflections	*l* = −14→14
4915 independent reflections	

### trans-Diaquabis(propane-1,3-diamine-κ2N,N')nickel(II) naphthalene-1,5-disulfonate (I) Refinement

**Table:**

Refinement on *F*^2^	Primary atom site location: structure-invariant direct methods
Least-squares matrix: full	Secondary atom site location: difference Fourier map
*R*[*F*^2^ > 2σ(*F*^2^)] = 0.024	Hydrogen site location: mixed
*wR*(*F*^2^) = 0.063	H atoms treated by a mixture of independent and constrained refinement
*S* = 1.05	*w* = 1/[σ^2^(*F*_o_^2^) + (0.0276*P*)^2^ + 0.4313*P*] where *P* = (*F*_o_^2^ + 2*F*_c_^2^)/3
4915 reflections	(Δ/σ)_max_ = 0.001
160 parameters	Δρ_max_ = 0.49 e Å^−^^3^
6 restraints	Δρ_min_ = −0.45 e Å^−^^3^

### trans-Diaquabis(propane-1,3-diamine-κ2N,N')nickel(II) naphthalene-1,5-disulfonate (I) Special details

**Table:**

Geometry. All esds (except the esd in the dihedral angle between two l.s. planes) are estimated using the full covariance matrix. The cell esds are taken into account individually in the estimation of esds in distances, angles and torsion angles; correlations between esds in cell parameters are only used when they are defined by crystal symmetry. An approximate (isotropic) treatment of cell esds is used for estimating esds involving l.s. planes.

### trans-Diaquabis(propane-1,3-diamine-κ2N,N')nickel(II) naphthalene-1,5-disulfonate (I) Fractional atomic coordinates and isotropic or equivalent isotropic displacement parameters (Å^2^)

**Table:**

	*x*	*y*	*z*	*U*_iso_*/*U*_eq_	
Ni1	0.500000	0.500000	0.000000	0.01186 (4)	
S1	0.70679 (2)	0.58894 (2)	0.49483 (2)	0.01321 (4)	
O1	0.66084 (6)	0.46643 (7)	0.45862 (9)	0.02052 (13)	
O2	0.64514 (6)	0.68190 (7)	0.39781 (9)	0.02174 (14)	
O3	0.71298 (6)	0.61736 (7)	0.65499 (8)	0.02114 (14)	
O1W	0.49621 (6)	0.41740 (7)	0.21368 (8)	0.01817 (12)	
H1W	0.5388 (13)	0.4416 (15)	0.2909 (15)	0.027*	
H2W	0.4313 (12)	0.4063 (14)	0.2445 (17)	0.027*	
N1	0.44649 (8)	0.66749 (8)	0.08156 (10)	0.02049 (15)	
H1N	0.4992 (13)	0.6929 (14)	0.1609 (16)	0.031*	
H2N	0.4508 (15)	0.7228 (13)	0.0109 (16)	0.031*	
N2	0.32384 (8)	0.43843 (9)	−0.06461 (10)	0.02132 (16)	
H3N	0.3261 (15)	0.3583 (12)	−0.0453 (18)	0.032*	
H4N	0.3126 (15)	0.4430 (16)	−0.1632 (15)	0.032*	
C1	0.32692 (10)	0.67556 (9)	0.12968 (13)	0.0249 (2)	
H1A	0.310814	0.761263	0.155196	0.030*	
H1B	0.325386	0.625806	0.221760	0.030*	
C2	0.22970 (10)	0.63152 (11)	0.00790 (13)	0.0284 (2)	
H2A	0.244925	0.665585	−0.090222	0.034*	
H2B	0.152384	0.664235	0.029653	0.034*	
C3	0.21966 (9)	0.49362 (11)	−0.00668 (12)	0.02373 (19)	
H3A	0.211983	0.458221	0.093386	0.028*	
H3B	0.146609	0.473004	−0.075744	0.028*	
C4	0.85697 (7)	0.58798 (8)	0.45455 (9)	0.01372 (14)	
C5	0.88796 (8)	0.67320 (8)	0.35417 (10)	0.01628 (15)	
H5	0.831367	0.732016	0.312373	0.020*	
C6	1.00413 (8)	0.67325 (9)	0.31317 (11)	0.01825 (16)	
H6	1.025026	0.732127	0.243378	0.022*	
C7	1.08703 (8)	0.58934 (9)	0.37293 (10)	0.01694 (15)	
H7	1.164422	0.590372	0.343242	0.020*	
C8	1.05880 (7)	0.50074 (8)	0.47877 (9)	0.01321 (13)	

### trans-Diaquabis(propane-1,3-diamine-κ2N,N')nickel(II) naphthalene-1,5-disulfonate (I) Atomic displacement parameters (Å^2^)

**Table:**

	*U*^11^	*U*^22^	*U*^33^	*U*^12^	*U*^13^	*U*^23^
Ni1	0.01178 (7)	0.01213 (7)	0.01176 (7)	−0.00176 (5)	0.00194 (5)	−0.00112 (5)
S1	0.01056 (8)	0.01543 (9)	0.01360 (8)	0.00099 (6)	0.00144 (6)	−0.00184 (6)
O1	0.0148 (3)	0.0189 (3)	0.0267 (3)	−0.0022 (2)	−0.0015 (2)	−0.0050 (3)
O2	0.0152 (3)	0.0243 (3)	0.0259 (3)	0.0069 (3)	0.0031 (2)	0.0061 (3)
O3	0.0173 (3)	0.0314 (4)	0.0154 (3)	−0.0027 (3)	0.0045 (2)	−0.0069 (3)
O1W	0.0162 (3)	0.0241 (3)	0.0140 (3)	−0.0048 (2)	0.0012 (2)	0.0015 (2)
N1	0.0253 (4)	0.0150 (3)	0.0234 (4)	−0.0025 (3)	0.0115 (3)	−0.0018 (3)
N2	0.0165 (3)	0.0268 (4)	0.0205 (4)	−0.0055 (3)	0.0021 (3)	−0.0052 (3)
C1	0.0295 (5)	0.0168 (4)	0.0322 (5)	0.0016 (4)	0.0177 (4)	−0.0005 (4)
C2	0.0225 (5)	0.0319 (5)	0.0319 (5)	0.0109 (4)	0.0083 (4)	0.0117 (4)
C3	0.0149 (4)	0.0350 (5)	0.0213 (4)	−0.0035 (4)	0.0027 (3)	0.0004 (4)
C4	0.0112 (3)	0.0158 (3)	0.0142 (3)	0.0011 (3)	0.0019 (2)	−0.0007 (3)
C5	0.0145 (3)	0.0166 (4)	0.0176 (3)	0.0016 (3)	0.0014 (3)	0.0021 (3)
C6	0.0156 (4)	0.0192 (4)	0.0203 (4)	0.0000 (3)	0.0033 (3)	0.0059 (3)
C7	0.0129 (3)	0.0198 (4)	0.0186 (4)	0.0000 (3)	0.0040 (3)	0.0038 (3)
C8	0.0115 (3)	0.0146 (3)	0.0136 (3)	0.0002 (3)	0.0018 (2)	0.0002 (3)

### trans-Diaquabis(propane-1,3-diamine-κ2N,N')nickel(II) naphthalene-1,5-disulfonate (I) Geometric parameters (Å, º)

**Table:**

Ni1—N1^i^	2.0960 (9)	C1—C2	1.5197 (18)
Ni1—N1	2.0961 (9)	C1—H1A	0.9900
Ni1—N2^i^	2.1127 (9)	C1—H1B	0.9900
Ni1—N2	2.1127 (9)	C2—C3	1.5210 (18)
Ni1—O1W	2.1205 (7)	C2—H2A	0.9900
Ni1—O1W^i^	2.1205 (7)	C2—H2B	0.9900
S1—O2	1.4550 (7)	C3—H3A	0.9900
S1—O3	1.4583 (7)	C3—H3B	0.9900
S1—O1	1.4614 (8)	C4—C5	1.3739 (12)
S1—C4	1.7863 (9)	C4—C8^ii^	1.4353 (12)
O1W—H1W	0.831 (13)	C5—C6	1.4132 (13)
O1W—H2W	0.828 (13)	C5—H5	0.9500
N1—C1	1.4785 (13)	C6—C7	1.3710 (13)
N1—H1N	0.908 (13)	C6—H6	0.9500
N1—H2N	0.883 (13)	C7—C8	1.4227 (12)
N2—C3	1.4809 (14)	C7—H7	0.9500
N2—H3N	0.895 (13)	C8—C8^ii^	1.4341 (17)
N2—H4N	0.875 (13)		
			
N1^i^—Ni1—N1	180.0	H3N—N2—H4N	104.4 (15)
N1^i^—Ni1—N2^i^	93.92 (4)	N1—C1—C2	112.12 (9)
N1—Ni1—N2^i^	86.08 (4)	N1—C1—H1A	109.2
N1^i^—Ni1—N2	86.08 (4)	C2—C1—H1A	109.2
N1—Ni1—N2	93.92 (4)	N1—C1—H1B	109.2
N2^i^—Ni1—N2	180.0	C2—C1—H1B	109.2
N1^i^—Ni1—O1W	88.92 (3)	H1A—C1—H1B	107.9
N1—Ni1—O1W	91.08 (3)	C1—C2—C3	114.59 (9)
N2^i^—Ni1—O1W	91.33 (3)	C1—C2—H2A	108.6
N2—Ni1—O1W	88.67 (3)	C3—C2—H2A	108.6
N1^i^—Ni1—O1W^i^	91.08 (3)	C1—C2—H2B	108.6
N1—Ni1—O1W^i^	88.92 (3)	C3—C2—H2B	108.6
N2^i^—Ni1—O1W^i^	88.67 (3)	H2A—C2—H2B	107.6
N2—Ni1—O1W^i^	91.33 (3)	N2—C3—C2	112.49 (9)
O1W—Ni1—O1W^i^	180.0	N2—C3—H3A	109.1
O2—S1—O3	113.05 (5)	C2—C3—H3A	109.1
O2—S1—O1	112.59 (5)	N2—C3—H3B	109.1
O3—S1—O1	112.50 (5)	C2—C3—H3B	109.1
O2—S1—C4	106.12 (4)	H3A—C3—H3B	107.8
O3—S1—C4	106.16 (4)	C5—C4—C8^ii^	121.23 (8)
O1—S1—C4	105.70 (4)	C5—C4—S1	117.52 (7)
Ni1—O1W—H1W	122.2 (11)	C8^ii^—C4—S1	121.23 (6)
Ni1—O1W—H2W	119.3 (11)	C4—C5—C6	119.92 (8)
H1W—O1W—H2W	102.2 (15)	C4—C5—H5	120.0
C1—N1—Ni1	118.24 (6)	C6—C5—H5	120.0
C1—N1—H1N	106.6 (10)	C7—C6—C5	120.78 (8)
Ni1—N1—H1N	110.5 (10)	C7—C6—H6	119.6
C1—N1—H2N	108.0 (11)	C5—C6—H6	119.6
Ni1—N1—H2N	107.9 (10)	C6—C7—C8	120.94 (8)
H1N—N1—H2N	104.8 (14)	C6—C7—H7	119.5
C3—N2—Ni1	122.81 (7)	C8—C7—H7	119.5
C3—N2—H3N	109.7 (11)	C7—C8—C8^ii^	118.96 (10)
Ni1—N2—H3N	105.0 (11)	C7—C8—C4^ii^	122.89 (8)
C3—N2—H4N	108.3 (11)	C8^ii^—C8—C4^ii^	118.16 (9)
Ni1—N2—H4N	105.1 (11)		

Symmetry codes: (i) −*x*+1, −*y*+1, −*z*; (ii) −*x*+2, −*y*+1, −*z*+1.

### trans-Diaquabis(propane-1,3-diamine-κ2N,N')nickel(II) naphthalene-1,5-disulfonate (I) Hydrogen-bond geometry (Å, º)

**Table:**

*D*—H···*A*	*D*—H	H···*A*	*D*···*A*	*D*—H···*A*
O1*W*—H2*W*···O3^iii^	0.83 (1)	1.99 (1)	2.8095 (10)	173 (2)
O1*W*—H1*W*···O1	0.83 (1)	1.92 (1)	2.7324 (10)	167 (2)
N1—H1*N*···O2	0.91 (1)	2.51 (1)	3.3726 (13)	159 (1)
N2—H3*N*···O2^iv^	0.90 (1)	2.34 (1)	3.1817 (13)	157 (1)
N2—H4*N*···O2^i^	0.88 (1)	2.60 (2)	3.3207 (12)	140 (1)

Symmetry codes: (i) −*x*+1, −*y*+1, −*z*; (iii) −*x*+1, −*y*+1, −*z*+1; (iv) −*x*+1, *y*−1/2, −*z*+1/2.

### trans-Diaquabis(propane-1,3-diamine-κ2N,N')nickel(II) naphthalene-2,6-disulfonate (II) Crystal data

**Table:**

[Ni(C_3_H_10_N_2_)_2_(H_2_O)_2_](C_10_H_6_O_6_S_2_)	*Z* = 1
*M**_r_* = 529.27	*F*(000) = 278
Triclinic, *P*1	*D*_x_ = 1.588 Mg m^−^^3^
*a* = 7.9530 (3) Å	Mo *K*α radiation, λ = 0.71073 Å
*b* = 8.1103 (3) Å	Cell parameters from 9894 reflections
*c* = 9.7183 (3) Å	θ = 2.8–33.1°
α = 89.9982 (11)°	µ = 1.12 mm^−^^1^
β = 81.8907 (11)°	*T* = 150 K
γ = 63.368 (1)°	Parallelepiped, violet
*V* = 553.32 (3) Å^3^	0.44 × 0.20 × 0.12 mm

### trans-Diaquabis(propane-1,3-diamine-κ2N,N')nickel(II) naphthalene-2,6-disulfonate (II) Data collection

**Table:**

APEX duo with Photon II area detector diffractometer	3850 reflections with *I* > 2σ(*I*)
Radiation source: fine-focus sealed tube	*R*_int_ = 0.028
φ and ω scans	θ_max_ = 33.2°, θ_min_ = 2.1°
Absorption correction: multi-scan (SADABS; Krause *et al.*, 2015)	*h* = −12→12
*T*_min_ = 0.660, *T*_max_ = 0.747	*k* = −12→12
29051 measured reflections	*l* = −14→14
4211 independent reflections	

### trans-Diaquabis(propane-1,3-diamine-κ2N,N')nickel(II) naphthalene-2,6-disulfonate (II) Refinement

**Table:**

Refinement on *F*^2^	Primary atom site location: structure-invariant direct methods
Least-squares matrix: full	Secondary atom site location: difference Fourier map
*R*[*F*^2^ > 2σ(*F*^2^)] = 0.022	Hydrogen site location: mixed
*wR*(*F*^2^) = 0.058	H atoms treated by a mixture of independent and constrained refinement
*S* = 1.06	*w* = 1/[σ^2^(*F*_o_^2^) + (0.0266*P*)^2^ + 0.1786*P*] where *P* = (*F*_o_^2^ + 2*F*_c_^2^)/3
4211 reflections	(Δ/σ)_max_ < 0.001
160 parameters	Δρ_max_ = 0.46 e Å^−^^3^
6 restraints	Δρ_min_ = −0.40 e Å^−^^3^

### trans-Diaquabis(propane-1,3-diamine-κ2N,N')nickel(II) naphthalene-2,6-disulfonate (II) Special details

**Table:**

Geometry. All esds (except the esd in the dihedral angle between two l.s. planes) are estimated using the full covariance matrix. The cell esds are taken into account individually in the estimation of esds in distances, angles and torsion angles; correlations between esds in cell parameters are only used when they are defined by crystal symmetry. An approximate (isotropic) treatment of cell esds is used for estimating esds involving l.s. planes.

### trans-Diaquabis(propane-1,3-diamine-κ2N,N')nickel(II) naphthalene-2,6-disulfonate (II) Fractional atomic coordinates and isotropic or equivalent isotropic displacement parameters (Å^2^)

**Table:**

	*x*	*y*	*z*	*U*_iso_*/*U*_eq_	
Ni1	0.500000	0.500000	0.500000	0.01258 (4)	
S1	0.21076 (3)	0.14345 (3)	0.33755 (2)	0.01595 (5)	
O1	0.41712 (11)	0.07412 (12)	0.30570 (9)	0.02572 (16)	
O2	0.10953 (11)	0.34311 (10)	0.36511 (9)	0.02322 (15)	
O3	0.15378 (12)	0.04556 (12)	0.44533 (8)	0.02541 (16)	
O1W	0.61858 (11)	0.22987 (10)	0.40417 (8)	0.01870 (13)	
H1W	0.552 (2)	0.190 (2)	0.3720 (16)	0.028*	
H2W	0.684 (2)	0.149 (2)	0.4515 (15)	0.028*	
N1	0.70875 (12)	0.40258 (12)	0.63276 (9)	0.01801 (15)	
H1N	0.790 (2)	0.446 (2)	0.6081 (16)	0.027*	
H2N	0.783 (2)	0.2799 (18)	0.6154 (16)	0.027*	
N2	0.31602 (13)	0.43491 (13)	0.64373 (9)	0.02010 (15)	
H3N	0.1967 (19)	0.493 (2)	0.6252 (17)	0.030*	
H4N	0.354 (2)	0.3138 (18)	0.6294 (16)	0.030*	
C1	0.64342 (16)	0.44315 (14)	0.78519 (11)	0.02175 (18)	
H1A	0.584826	0.578454	0.807031	0.026*	
H1B	0.754942	0.384928	0.834190	0.026*	
C2	0.49963 (17)	0.37299 (14)	0.83887 (10)	0.02265 (19)	
H2A	0.553073	0.241033	0.806376	0.027*	
H2B	0.482087	0.380444	0.941971	0.027*	
C3	0.30612 (16)	0.47729 (14)	0.79356 (11)	0.02272 (19)	
H3A	0.213780	0.442867	0.849048	0.027*	
H3B	0.260449	0.611846	0.811525	0.027*	
C4	0.01803 (13)	0.01806 (12)	0.18912 (10)	0.01534 (15)	
H4	−0.030957	−0.008594	0.276542	0.018*	
C5	0.14324 (13)	0.09259 (12)	0.18246 (10)	0.01522 (15)	
C6	0.22107 (15)	0.13071 (14)	0.05315 (11)	0.02002 (18)	
H6	0.308291	0.181511	0.050351	0.024*	
C7	0.16995 (14)	0.09397 (14)	−0.06789 (11)	0.01973 (17)	
H7	0.222939	0.118660	−0.154552	0.024*	
C8	0.03830 (12)	0.01922 (12)	−0.06491 (9)	0.01458 (15)	

### trans-Diaquabis(propane-1,3-diamine-κ2N,N')nickel(II) naphthalene-2,6-disulfonate (II) Atomic displacement parameters (Å^2^)

**Table:**

	*U*^11^	*U*^22^	*U*^33^	*U*^12^	*U*^13^	*U*^23^
Ni1	0.01234 (7)	0.01358 (7)	0.01365 (7)	−0.00721 (6)	−0.00296 (5)	0.00048 (5)
S1	0.01467 (10)	0.01617 (10)	0.01876 (10)	−0.00778 (8)	−0.00518 (8)	−0.00098 (7)
O1	0.0149 (3)	0.0302 (4)	0.0318 (4)	−0.0095 (3)	−0.0054 (3)	−0.0091 (3)
O2	0.0233 (3)	0.0175 (3)	0.0287 (4)	−0.0085 (3)	−0.0061 (3)	−0.0044 (3)
O3	0.0320 (4)	0.0310 (4)	0.0225 (4)	−0.0202 (3)	−0.0123 (3)	0.0083 (3)
O1W	0.0194 (3)	0.0156 (3)	0.0227 (3)	−0.0083 (3)	−0.0072 (3)	−0.0005 (2)
N1	0.0186 (4)	0.0183 (3)	0.0185 (4)	−0.0086 (3)	−0.0061 (3)	0.0002 (3)
N2	0.0172 (4)	0.0224 (4)	0.0223 (4)	−0.0104 (3)	−0.0026 (3)	0.0038 (3)
C1	0.0303 (5)	0.0198 (4)	0.0176 (4)	−0.0119 (4)	−0.0096 (4)	0.0008 (3)
C2	0.0338 (5)	0.0180 (4)	0.0159 (4)	−0.0112 (4)	−0.0050 (4)	0.0023 (3)
C3	0.0246 (5)	0.0183 (4)	0.0204 (4)	−0.0076 (4)	0.0041 (4)	0.0001 (3)
C4	0.0144 (4)	0.0157 (4)	0.0169 (4)	−0.0075 (3)	−0.0032 (3)	0.0000 (3)
C5	0.0141 (4)	0.0142 (3)	0.0184 (4)	−0.0067 (3)	−0.0046 (3)	−0.0006 (3)
C6	0.0213 (4)	0.0246 (4)	0.0217 (4)	−0.0165 (4)	−0.0047 (3)	0.0022 (3)
C7	0.0210 (4)	0.0260 (4)	0.0193 (4)	−0.0168 (4)	−0.0035 (3)	0.0022 (3)
C8	0.0132 (3)	0.0143 (3)	0.0179 (4)	−0.0074 (3)	−0.0031 (3)	0.0003 (3)

### trans-Diaquabis(propane-1,3-diamine-κ2N,N')nickel(II) naphthalene-2,6-disulfonate (II) Geometric parameters (Å, º)

**Table:**

Ni1—O1W^i^	2.1110 (7)	C1—C2	1.5227 (16)
Ni1—O1W	2.1110 (7)	C1—H1A	0.9900
Ni1—N1	2.1151 (8)	C1—H1B	0.9900
Ni1—N1^i^	2.1151 (8)	C2—C3	1.5171 (16)
Ni1—N2	2.1181 (9)	C2—H2A	0.9900
Ni1—N2^i^	2.1181 (9)	C2—H2B	0.9900
S1—O2	1.4534 (8)	C3—H3A	0.9900
S1—O3	1.4565 (8)	C3—H3B	0.9900
S1—O1	1.4612 (8)	C4—C5	1.3720 (12)
S1—C5	1.7769 (9)	C4—C8^ii^	1.4224 (13)
O1W—H1W	0.829 (13)	C4—H4	0.9500
O1W—H2W	0.823 (13)	C5—C6	1.4177 (14)
N1—C1	1.4839 (13)	C6—C7	1.3717 (14)
N1—H1N	0.871 (13)	C6—H6	0.9500
N1—H2N	0.901 (12)	C7—C8	1.4231 (12)
N2—C3	1.4792 (14)	C7—H7	0.9500
N2—H3N	0.895 (13)	C8—C8^ii^	1.4189 (18)
N2—H4N	0.893 (13)		
			
O1W^i^—Ni1—O1W	180.00 (5)	H3N—N2—H4N	106.6 (14)
O1W^i^—Ni1—N1	91.08 (3)	N1—C1—C2	112.55 (8)
O1W—Ni1—N1	88.92 (3)	N1—C1—H1A	109.1
O1W^i^—Ni1—N1^i^	88.92 (3)	C2—C1—H1A	109.1
O1W—Ni1—N1^i^	91.08 (3)	N1—C1—H1B	109.1
N1—Ni1—N1^i^	180.0	C2—C1—H1B	109.1
O1W^i^—Ni1—N2	90.87 (3)	H1A—C1—H1B	107.8
O1W—Ni1—N2	89.13 (3)	C3—C2—C1	114.71 (8)
N1—Ni1—N2	90.30 (3)	C3—C2—H2A	108.6
N1^i^—Ni1—N2	89.70 (3)	C1—C2—H2A	108.6
O1W^i^—Ni1—N2^i^	89.13 (3)	C3—C2—H2B	108.6
O1W—Ni1—N2^i^	90.87 (3)	C1—C2—H2B	108.6
N1—Ni1—N2^i^	89.70 (3)	H2A—C2—H2B	107.6
N1^i^—Ni1—N2^i^	90.30 (3)	N2—C3—C2	111.08 (8)
N2—Ni1—N2^i^	180.0	N2—C3—H3A	109.4
O2—S1—O3	112.75 (5)	C2—C3—H3A	109.4
O2—S1—O1	112.81 (5)	N2—C3—H3B	109.4
O3—S1—O1	112.93 (5)	C2—C3—H3B	109.4
O2—S1—C5	106.41 (4)	H3A—C3—H3B	108.0
O3—S1—C5	106.06 (4)	C5—C4—C8^ii^	119.89 (8)
O1—S1—C5	105.10 (4)	C5—C4—H4	120.1
Ni1—O1W—H1W	121.5 (11)	C8^ii^—C4—H4	120.1
Ni1—O1W—H2W	114.1 (11)	C4—C5—C6	121.22 (8)
H1W—O1W—H2W	105.6 (15)	C4—C5—S1	120.11 (7)
C1—N1—Ni1	117.93 (7)	C6—C5—S1	118.68 (7)
C1—N1—H1N	108.4 (10)	C7—C6—C5	119.74 (8)
Ni1—N1—H1N	108.3 (10)	C7—C6—H6	120.1
C1—N1—H2N	108.7 (10)	C5—C6—H6	120.1
Ni1—N1—H2N	110.4 (10)	C6—C7—C8	120.64 (9)
H1N—N1—H2N	101.8 (14)	C6—C7—H7	119.7
C3—N2—Ni1	117.46 (7)	C8—C7—H7	119.7
C3—N2—H3N	106.8 (10)	C8^ii^—C8—C4^ii^	119.26 (10)
Ni1—N2—H3N	110.3 (10)	C8^ii^—C8—C7	119.24 (10)
C3—N2—H4N	108.5 (10)	C4^ii^—C8—C7	121.50 (8)
Ni1—N2—H4N	106.7 (10)		

Symmetry codes: (i) −*x*+1, −*y*+1, −*z*+1; (ii) −*x*, −*y*, −*z*.

### trans-Diaquabis(propane-1,3-diamine-κ2N,N')nickel(II) naphthalene-2,6-disulfonate (II) Hydrogen-bond geometry (Å, º)

**Table:**

*D*—H···*A*	*D*—H	H···*A*	*D*···*A*	*D*—H···*A*
O1*W*—H1*W*···O1	0.83 (1)	1.88 (1)	2.7065 (10)	174 (2)
O1*W*—H2*W*···O3^iii^	0.82 (1)	1.92 (1)	2.7437 (11)	177 (2)
N1—H1*N*···O2^i^	0.87 (1)	2.22 (1)	3.0039 (11)	150 (1)
N1—H2*N*···O3^iii^	0.90 (1)	2.51 (1)	3.3444 (12)	155 (1)
N2—H3*N*···O2^iv^	0.90 (1)	2.18 (1)	3.0533 (12)	166 (1)

Symmetry codes: (i) −*x*+1, −*y*+1, −*z*+1; (iii) −*x*+1, −*y*, −*z*+1; (iv) −*x*, −*y*+1, −*z*+1.
